# Report of the Board of Visitors of the Boston Lunatic Asylum, in the Matter of the Superintendent of That Institution

**Published:** 1851-01-01

**Authors:** 


### REPORT OF THE BOARD OF VISITORS OF THE BOSTON LUNATIC ASYLUM, IN THE MATTER OF THE SUPERINTENDENT OF THAT INSTITUTION.—Boston, 1849.

It appears that *Dr. C. H. Stedman* is the physician to the *Boston Lunatic Asylum;* that in the month of December, 1849, he was called upon by a particular professional friend, in his private character, to see a *Mrs. Helen Kraitser*, the wife of a physician of that name; and, after instituting a proper examination into her state of mind, signed a certificate of her insanity. It appears that the *Board of Visitors of the Boston Lunatic Hospital* received instructions from the *City Council* to institute an investigation into the matter, and to request their medical officer (Dr. Stedman), as well as other parties connected with the transaction, to make a statement, in writing, of such facts bearing upon the matter as they pleased to communicate. Dr. Stedman, with a very commendable spirit, denies the right of the Board of Visitors, at the instigation of the Common Council, to call upon him to explain any act of his, performed in his *private character as a physician, quite unconnected with his public duties as medical superintendent of the Boston Hospital.* The certificate in question was signed by Dr. Stedman, not as the physician of a public asylum, nor for the purpose of being used there. He exclaims, and that with perfect justness, against the doctrine, that individuals holding public appointments are to be subjected to arraignment and trial by the legislative branch of a municipal government, upon charges or insinuations of criminal offences, alleged or supposed to have been committed by them in their *private capacities*, and not in the discharge of their *public duties.* Notwithstanding, however, this temperate protest against what we conceive to have been an unwarrantable interference on the part of the Common Council with the private act of Dr. Stedman, that physician laid a statement of the facts of the case before the Board of Visitors; and they placed themselves, we think improperly, in communication with the patient herself. Mrs. Kraister directed the Board to her solicitors; and the solicitors, after a little coquetry, intimate to the Visitors, that Mrs. Kraister, acting on advice, declines sending the proposed statement—and there, we presume, the matter rests.

---

